# Explaining Away the Body: Experiences of Supernaturally Caused Touch and Touch on Non-Hand Objects within the Rubber Hand Illusion

**DOI:** 10.1371/journal.pone.0009416

**Published:** 2010-02-24

**Authors:** Jakob Hohwy, Bryan Paton

**Affiliations:** 1 Philosophy Program, Monash University, Clayton, Victoria, Australia; 2 School of Psychology and Psychiatry, Monash University, Clayton, Victoria, Australia; University of Barcelona, Spain

## Abstract

**Background:**

In rubber hand illusions and full body illusions, touch sensations are projected to non-body objects such as rubber hands, dolls or virtual bodies. The robustness, limits and further perceptual consequences of such illusions are not yet fully explored or understood. A number of experiments are reported that test the limits of a variant of the rubber hand illusion.

**Methodology/Principal Findings:**

A variant of the rubber hand illusion is explored, in which the real and foreign hands are aligned in personal space. The presence of the illusion is ascertained with participants' scores and temperature changes of the real arm. This generates a basic illusion of touch projected to a foreign arm. Participants are presented with further, unusual visuotactile stimuli subsequent to onset of the basic illusion. Such further visuotactile stimulation is found to generate very unusual experiences of supernatural touch and touch on a non-hand object. The finding of touch on a non-hand object conflicts with prior findings, and to resolve this conflict a further hypothesis is successfully tested: that without prior onset of the basic illusion this unusual experience does not occur.

**Conclusions/Significance:**

A rubber hand illusion is found that can arise when the real and the foreign arm are aligned in personal space. This illusion persists through periods of no tactile stimulation and is strong enough to allow very unusual experiences of touch felt on a cardboard box and experiences of touch produced at a distance, as if by supernatural causation. These findings suggest that one's visual body image is explained away during experience of the illusion and they may be of further importance to understanding the role of experience in delusion formation. The findings of touch on non-hand objects may help reconcile conflicting results in this area of research. In addition, new evidence is provided that relates to the recently discovered psychologically induced temperature changes that occur during the illusion.

## Introduction

In an intriguing type of illusion, touch sensations can be felt as produced on objects, such as rubber hands, mannequins or virtual bodies, located away from participants' real limbs or bodies [1], [2], [3], [4], [5], [6]. These illusions arise in an effort to integrate conflicting visuotactile stimuli, and this process can override prior knowledge of the visual body-image, proprioception or self-location, as well as general background knowledge.

To gain a better understanding of such phenomena is it desirable to investigate their robustness, limits and their consequences for further sensory processing. It is therefore investigated whether, subsequent to the onset of such an illusion, unusual visuotactile stimuli are incorporated into them, that is, whether there can be further visuotactile illusions within a version of the rubber hand illusion. This is done utilising a limb-specific variation of a paradigm used for full-body illusions (FBI) [7].

A broadly probabilistic approach can be taken to many types of illusions [8]. Probabilistic cognitive processes would occur mostly at a sub-personal level but can be described in terms borrowed from philosophy of science: illusions arise as the system in question seeks the best explanation or model of the sensory input. In the standard RHI the sensory input to be explained comprises the touch produced synchronously or asynchronously with a visual stimulus, the visual input of the clearly artificial rubber hand, and the proprioceptive incongruence between one's own hand and the viewed rubber hand. There is also some background knowledge, namely of the experimental set-up, of one's visual body image, of the nature of causal relations in general, and the general fact that rubber hands cannot feel touch. The intriguing fact about the RHI is that synchronous touch, which weighs in favour of projecting touch to the rubber hand, can dominate the other kinds of evidence, all of which weigh against projecting touch to the rubber hand.

**Figure 1:**
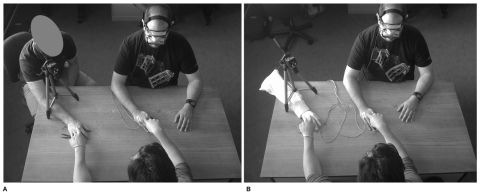
Experiment set-up viewed from above, showing relative locations of camera, experimenters and participant. A. Experimental set up for experiment 1. B. Experimental set up for experiment 2 and 3. The Participant is seated to the right, wearing goggles. Experimenter A is seated opposite the participant. Experimenter B is seated on the left in A. A rubber hand is used in B.

Given this approach, a situation with less proprioceptive incongruence is likely to strengthen and stabilise the illusion and ensure fast onset (consistent also with Ref. [9] which showed illusion strength to decrease with increasing proprioceptive disparity). Therefore, a variation of the recently discovered full-body illusion is used here. Participants wearing head-mounted displays visually perceive touch in a location in virtual personal space (namely a finger moving in view of a remote camera as if poking the chest) that appears congruent with the location in personal space where they feel but do not actually see the tactile stimulus (namely by being poked on the chest) [3]. This contrasts with both the standard RHI and another version of the FBI in which participants see a touch being produced in peripersonal or extrapersonal space some distance from the limb's or body's known position in personal space [4]. In the set up used herein participants see an experimenter's real arm or an artificial arm in a head-mounted display and this arm appears to be aligned with their own arm in personal space (Figure 1). This set up differs from FBI by only concerning a specific limb. It differs from the standard RHI by aligning the real and the foreign arm in visual space, thereby eliminating proprioceptive discrepancy. Presence of the basic illusion where touch is projected to the seen foreign arm and felt as if produced by the visible finger tapping the foreign arm is here determined by participant scores as well as, as an independent measure, psychologically induced temperature changes of the participant's experimental limb [10]. This temperature measure is used here for the first time after its initial publication as an objective measure of a version of the RHI. The original findings of these temperature changes showed significant cooling of the real arm (as opposed to the contralateral arm or ipsilateral foot) during synchronous but not asynchronous touch. It was also found that the stronger the subjective ratings of the RHI, the stronger the temperature change [10]. This measure is used because in this design, where there is no proprioceptive discrepancy, the most commonly used measure of proprioceptive drift cannot be used.

Continuing the probabilistic approach, a rubber hand illusion can be considered the result of a perceptual process that provides the best explanation, given the currently available evidence, of the sensory input. This new, but illusionary, multisensory solution should then inform subsequent perceptual processing and undermine the previous body image [11]. We therefore expected that if further sensory input was introduced subsequent to illusion onset, then it would have a tendency to be incorporated into the illusion rather than work against and perhaps extinguish the illusion. This should lead to unusual experiences as further visuotactile conflict is resolved on the basis of false bodily self-representation.

The primary hypothesis is thus that there will be a rubber hand illusion during synchronous touch but not asynchronous touch in the basic set up, and that there will be odd and unusual experiences during continued synchronous touch after illusion onset as opposed to continued asynchronous touch. Support is found for this hypothesis as touch is found to be projected to non-hand objects and to be experienced as caused by supernatural means.

The finding of an illusion of touch felt on a non-hand object is relevant for addressing some conflicting findings concerning RHI-like illusions for non-hand objects. Armel and Ramachandran [1] developed the rubber hand illusion (RHI) [5] in unexpected ways such that participants projected a sensation of touch not only to a rubber hand but also to a bare table top and to a rubber hand located in extrapersonal space. Tsakiris and Haggard [6], [12] in contrast failed to establish the illusion of projected touch to a non-hand rubber object. To address this issue, we tested the secondary hypothesis that a RHI-like illusion for a non-hand object will not reliably occur when the participant is not already in the basic illusion. We find that without prior onset of the basic illusion there is no significant difference between reports of a RHI-like illusion for the non-hand object during synchronous touch vs. asynchronous touch. This suggests possible ways to reconcile the conflicting findings.

## Methods

### Participants

*Experiment 1:* 13 (8 male) healthy volunteers (age *M* = 36.123, *SD* = 12.08 years), 10 right handed. *Experiment 2*, *condition 1*: 11 (5 male) new healthy volunteers (age *M* = 26.0, *SD* = 8.01 years), 10 right handed; *condition 2*: 10 (3 male) new healthy volunteers (age *M* = 34.22 , *SD* = 16.15 years), 9 right handed. *Experiment 3:* 9 (4 male) new healthy volunteers (age *M* = 27.6, *SD* = 12.23 years), 8 right handed. All participants gave written, informed consent to participate in the study.

The study protocol was approved by the Monash University Human Research Ethics committee (CF09/0495 – 2009000183).

### Materials

#### Experimental setup

Participants wore a set of stereoscopic (dual input) OLED head mounted display (eMagin Z800) connected to a colour CCD camera (Sony CCD sensor, 480 Lines) mounted on a tripod. Participants sat opposite experimenter A (Figure 1). The view in the head mounted display was of experimenter B's right, lower arm and hand, or a rubber hand, or a cardboard box situated on a desk. The foreign arm (either Experimenter B's real arm or the rubber hand) was positioned to appear in the head mounted display to be spatially coincidental with the participant's own right arm and hand, which was lying upon the same desk (participants were free to move their arm until it was felt to be in the same position as the viewed foreign arm, after which movement was not permitted). Participants' view was also visible on a monitor viewed by the experimenters. Participants were aware of the set up. Tapping (*ca.* 1 tap/sec) on the forearm, near the wrist, was either synchronous or asynchronous; in the asynchronous condition there was a difference between seen and felt touch of *ca.* 500ms-1s, consistent with findings in [13]. For all experiments, synchronous and asynchronous tapping was counter-balanced across participants for all conditions. For experiments 2 & 3 question order was randomised.

#### Temperature measurements

**Figure 2:**
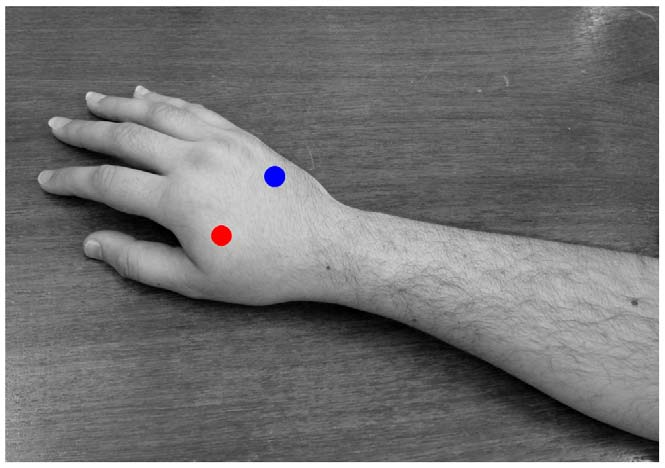
Temperature sensor placement on participant's hand.

The skin temperature (Thermistor, Murata NTH4G) of participants was recorded (2 Hz sampling rate) to assess how changes in skin temperature were related to presence or absence of the illusion in different conditions [10]. Temperature data were the average recorded data from two sites on the right hand of each participant (the experimental hand; see Figure 2); all temperature measures are in degrees Celsius. Skin temperature differences in the synchronous vs. asynchronous conditions were used as an independent measure of the general validity of this design. The often used measure of proprioceptive drift [6] cannot be used for cases where the illusory limb and the real limb appear to be aligned in personal space.

#### Questionnaire

Participants were asked to score their answer to a series of questions on a scale ranging from −3 to +3, zero inclusive that were designed to probe their experiences. A +3 rating was used to indicate a very strong affirmative answer, −3 was used to indicate a very strong negative answer; intermediate scores were used to indicate degrees of affirmation. All questions are listed in Table 1 and detailed below. In experiment 1, to ensure illusion onset, participants were asked to score orally the presence of the illusion after a short period of tapping and then again after a longer period of tapping (periods described below). In experiments 2 and 3, to avoid possible bias, questions were presented in writing after termination of each condition, and participants gave their score by drawing a line through the scale.

**Table 1:** Questions asked of and scored by participants across each experiment.

		Questions rated by participants
Experiment	Condition	Questions
1	1	1. “Is it as if the touch you can feel is produced by the finger you can see and on the arm you can see?”†
	2	1. “Is it as if the touch you can feel is produced by the finger you can see and on the arm you can see?”†
2	1	1. “While the finger was elevated off the rubber hand, was it as if the finger you could see in the goggles was causing the touch you could feel, even though there was a visible gap between the finger and the rubber arm?”†
		2. “Did the touch you could feel become painful?”‡
	2	1. “While you could see the box, was it as if the finger you could see in the goggles was causing a touch sensation on the box?”†
		2. “Did the touch you could feel become painful?”‡
		3. “Did it feel as if you had two bodies?”‡
3	1	1. “While you could see the box, was it as if the finger you could see in the goggles was causing a touch sensation on the box?”†
		2. “Did the touch you could feel become painful?”‡
		3. “Did it feel as if you had two bodies?”‡

† Questions used in the analysis.

‡ Control questions.

### Procedure

#### Experiment 1: basic illusion

Condition 1 in experiment 1 was a 2×2 repeated measures design; condition 2 in experiment 1 was a 2×4 repeated measures design. For both conditions the first factor was touch type (synchronous or asynchronous), this factor was within subjects, and counterbalanced. The second factor for both conditions was time. For the first condition the time factor was initial and 30s, for the second condition it was initial, 10s, 30s and 60s.

#### Experiment 1, condition 1: basic illusion during continuous touch

**Figure 3:**
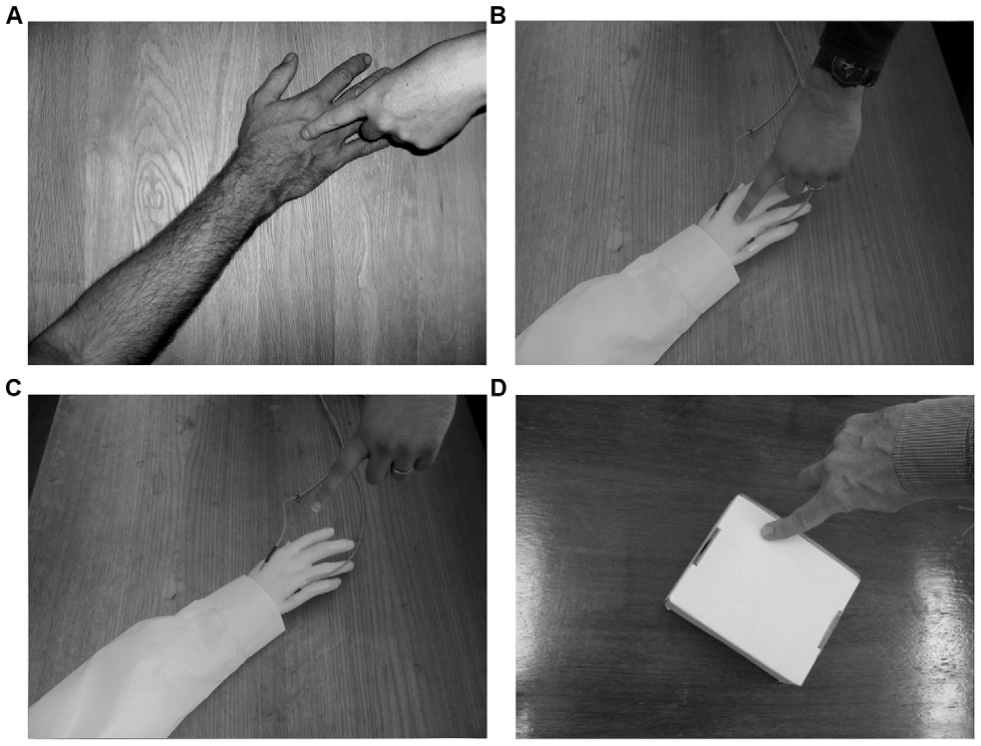
Examples of experimental conditions: participant's visual perspective. All concurrent with synchronous or asynchronous touch on participant's real, unseen arm. A) Moving, visible finger seen to touch visible foreign arm (experiment 1, condition 1 and 2). B) Moving visible finger seen to touch visible foreign rubber arm (experiment 2). C) Moving visible finger elevated off visible foreign rubber arm (experiment 2, condition 1). D) Moving visible finger seen to touch white box (experiment 2 and 3).

The initial illusion was induced by tapping either synchronously or asynchronously (with experimenter A's finger) the participant's actual forearm and the foreign forearm they could see in the head mounted display (Figure 3a). Participants were asked, after 10–20 seconds, to give a scored answer (rated on the 7-point scale) to the following question: “Is it as if the touch you can feel is produced by the finger you can see and on the arm you can see?” After 30 seconds of continuous tapping participants were asked to again rate their agreement with the same statement. Data from these two occurrences of the same one question were included in the analysis.

#### Experiment 1, condition 2: basic illusion during intermittent touch

This condition began with the same either synchronous or asynchronous initial tapping for 10–20 seconds and scoring to the same question as in condition 1 (Figure 3a). Immediately after the first scoring, consecutive periods of 10, then 30, then 60 seconds with no tapping were introduced. Participants would not be tapped during these periods and would see only the foreign arm in the head mounted display. At the end of each wait period, a single tap (in either synchrony or asynchrony depending on condition) was applied to both the real and the foreign arm and scores were again elicited. This condition lasted in total approx. 1 minute 50 seconds. Data from these four occurrences of the same one question were included in the analysis.

#### Experiment 2: supernaturally caused (elevated) touch and touch on non-hand objects

This experiment is a 2×2 mixed design with the first factor being touch type (synchronous or asynchronous, counterbalanced), this factor was within subjects, and counterbalanced. The second factor was condition, touch seen to be elevated off the rubber hand or touch on a cardboard box, this factor was between subjects.

#### Experiment 2, condition 1: supernaturally caused (elevated) touch

Both the synchronous and asynchronous tapping conditions began with 60 seconds of continuous tapping on the real arm and a rubber hand (Figure 3b). Then the visible, still moving finger was elevated approx. 5 cm off the visible rubber arm (Figure 3c) while touch continued for another 3 minutes on the real arm. Scores were elicited only after each condition had terminated. Participants were asked to score their answer to “While the finger was elevated off the rubber hand, was it as if the finger you could see in the goggles was causing the touch you could feel, even though there was a visible gap between the finger and the rubber arm?” Data from this one question was included in the final analysis. Participants also scored a further control question “Did the touch you could feel become painful?” At the conclusion of the experiment, participants were encouraged to give any open-ended descriptions of the experience they could offer (open ended descriptions encourage participants to describe their experience in their own words, participants typically write a couple of sentences).

#### Experiment 2, condition 2: touch felt on non-hand objects

Both the synchronous and asynchronous tapping conditions began with 30 seconds of continuous tapping on the real hand and a rubber hand. Then the view through the head mounted display was swapped (by switching to another camera) to touch of a small white cardboard box (Figure 3d). Tapping on the foreign arm and on the box continued for another 2 minutes and 30 seconds. Scores were elicited after each condition had terminated. Participants were asked to score their answer to “While you could see the box, was it as if the finger you could see in the goggles was causing a touch sensation on the box?” Data from this question was included in the final analysis. Participants also scored two further control questions “Did the touch you could feel become painful?” and “Did it feel as if you had two bodies?”

#### Experiment 3: touch felt on non-hand object with no prior onset of the basic illusion

Experiment 3 was a within subjects design with one factor, touch type (synchronous or asynchronous, counterbalanced). Both the synchronous and asynchronous tapping conditions consisted of 3 minutes of continuous tapping on the real hand and on a visible white cardboard box (Figure 3c). Scores were elicited after each condition had terminated. Participants were asked to score their answer to “While you could see the box, was it as if the finger you could see in the goggles was causing a touch sensation on the box?” Participants also scored two further control questions “Did the touch you could feel become painful?” and “Did it feel as if you had two bodies?”

### Data Analysis

#### Temperature data

For condition 1 of Experiment 1, data from a period of 30 seconds, beginning after the first scoring, were averaged to obtain a single temperature measurement for synchronous and asynchronous tapping for each participant. For condition 2, the last minute of temperature measurements were averaged to obtain a single temperature measurement for each participant for each tapping condition. For Experiment 2, 2 minutes and 30 seconds of temperature measurements, recorded after the onset period, were averaged to obtain a single temperature measurement for each participant for each tapping condition. For Experiment 3, 2 minutes of temperature measurements, recorded after the onset period, were averaged to obtain a single temperature measurement for each participant for each tapping condition. The data were analysed using paired samples t-tests (with bonferroni corrections) comparing synchronous tapping data to asynchronous tapping data.

#### Questionnaire data

For experiment 1, two way repeated measures ANOVAs with the first factor, tapping (synchronous, asynchronous) were used for the analysis of all conditions with only the second factor and its number of levels differing (see above). In experiment 1, each condition was analysed with a separate ANOVA. Even though we were not interested in comparisons between the two conditions in experiment 1 bonferroni corrections were nonetheless applied to each of the main effects and interactions to account for possible Type I error rate inflation. In experiment 2 a two way mixed model ANOVA with the first factor, tapping (synchronous, asynchronous, WS) and second factor condition (elevated touch, touch on a box, BS) was used for the analysis. In experiment 3 a paired samples t-test was used to analyse the data. In all experiments bonferroni corrected t-tests were used to analyse interaction effects as needed. Where control questions were included (experiment 2, conditions 1 & 2 and experiment 3, see Table 1) they were analysed using paired samples t-tests to see if there was any difference in scores between synchronous and asynchronous conditions. No significant differences were found and so the control questions were excluded from further analyses such that only the questions gauging the illusion were included in the final analyses.

## Results

### Experiment 1: Basic Illusion

#### Temperature data

**Figure 4:**
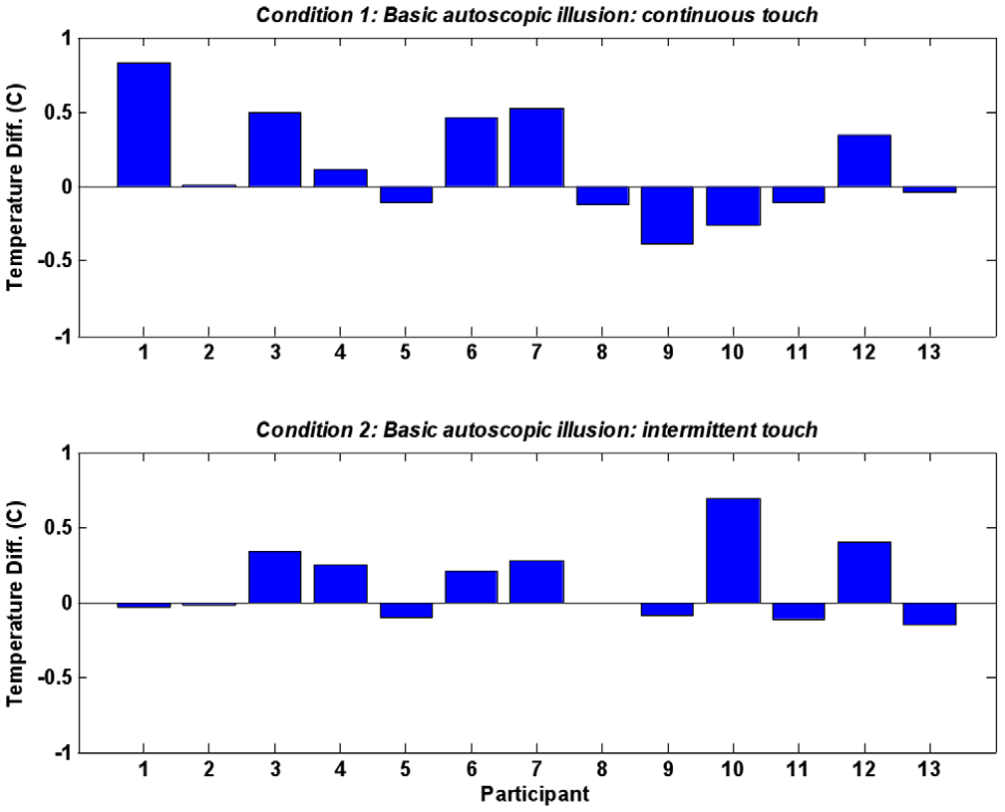
Temperature differences between synchronous and asynchronous touch. Condition 1 and condition 2 temperature differences between synchronous and asynchronous touch taken as the mean of the last 30 seconds of temperature data for condition 1 and the mean of the last 60 seconds of temperature data for condition 2. Positive temperature differences indicate higher temperatures in the asynchronous tapping condition.

In the original report of limb-specific temperature changes during the RHI [10] temperature changes were measured for approximately 5–8 mins only after participants reported illusion onset or after 5 mins of tapping. Accordingly, a paired samples t-test was here used to compare the two tapping conditions in condition 2 of Experiment 1 over a period of 60 seconds beginning approximately 50 seconds after tapping commenced), *t* (12) = −1.84, *p* = .04. Temperatures in the synchronous tapping condition (*M* = 28.92, *SD* = 2.27) were found to be, on average, 0.13 degrees lower than the asynchronous tapping condition temperatures (*M* = 29.05, *SD* = 2.31) (Figure 4). These data suggest that like [10], temperature change can be used as a physiological marker of the presence of a RHI using the present design in which the seen and real hand are spatially coincidental. The same method for inducing the basic illusion was therefore used in experiment 2 and 3. To further explore these temperature changes, a paired samples t-test was used to compare mean temperature measurements, in condition 1, for 30 seconds immediately after onset of continuous tapping, *t* (12) = −1.36, *p*>.09. Consistent with findings in [10], the temperature changes did not manifest at this early stage of tapping.

#### Questionnaire data

**Figure 5:**
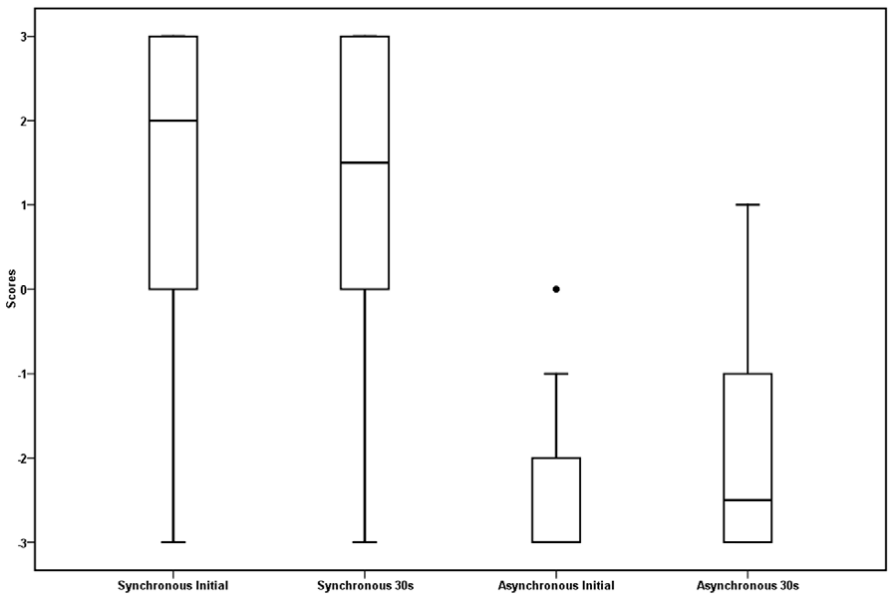
Box plots for condition 1, experiment 1. Box plots of scores for condition 1, experiment 1, initial and 30 s for synchronous and asynchronous tapping.

In Condition 1, Basic illusion during continuous touch, the presence and initial persistence of the illusion was investigated. A two way repeated measures ANOVA of the condition 1 data revealed a main effect of tapping type, *F* (1, 17) = 72.90, *p*<.01, such that participant's scores were higher (more affirmative) in the synchronous condition (*M* = 1.04, *SD* = 2.46) than in the asynchronous condition (*M* = −2.50, *SD* = 1.14) (see Table 1 for the question used in this analysis). There was no significant main effect of time (*p's*>.05) or interaction between time and tapping condition. These results indicate that the illusion was relatively robust as compared to the asynchronous tapping condition. These results support the described basic paradigm (Figure 1a and Figure 3a) as useful for inducing this version of the rubber hand illusion (Figure 5).

**Figure 6:**
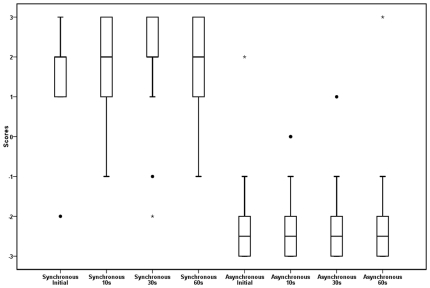
Box plots for condition 2, experiment 1. Box plots of scores for condition 2, experiment 1, initial, 10s, 30 s and 60 s for synchronous and asynchronous tapping.

In Condition 2, Basic illusion during intermittent touch, the robustness of the illusion under conditions with more minimal, intermittent tactile stimulation was tested. Ratings for the three wait periods (of 10, 30, and 60 seconds respectively) in condition 2 were compared for synchronous versus asynchronous tapping. A two way repeated measures ANOVA revealed a similar pattern as for condition 1 data with a main effect of touch type , *F* (1, 17) = 135.06, *p*<.01, with participants reporting higher affirmative scores in the synchronous condition (*M* = 1.81, *SD* = 1.02) than in the asynchronous condition (*M* = −2.19, *SD* = 1.10) (Figure 6) (see Table 1 for the question used in this analysis). There was no significant main effect of time or an interaction (*p's*>.05). These data reaffirm the utility of the paradigm and indicate that continuous stimulation is not necessary to maintain the illusion over the periods tested.

### Experiment 2: Supernaturally Caused (Elevated) Touch and Touch on Non-Hand Objects

In this experiment, scores of the presence of unusual experiences were elicited after the conclusion of each condition, to avoid bias. In addition, a rubber arm was used instead of, as in experiment 1 an experimenter's real arm, to make sure that the illusions we report also work for a clearly foreign, artificial arm. Lastly, concerning the temperature changes, it is not known how and if psychologically induced temperature changes are affected in these more unusual visuotactile conditions so we merely report, but have no hypothesis for temperature data for these further experiments.

**Figure 7:**
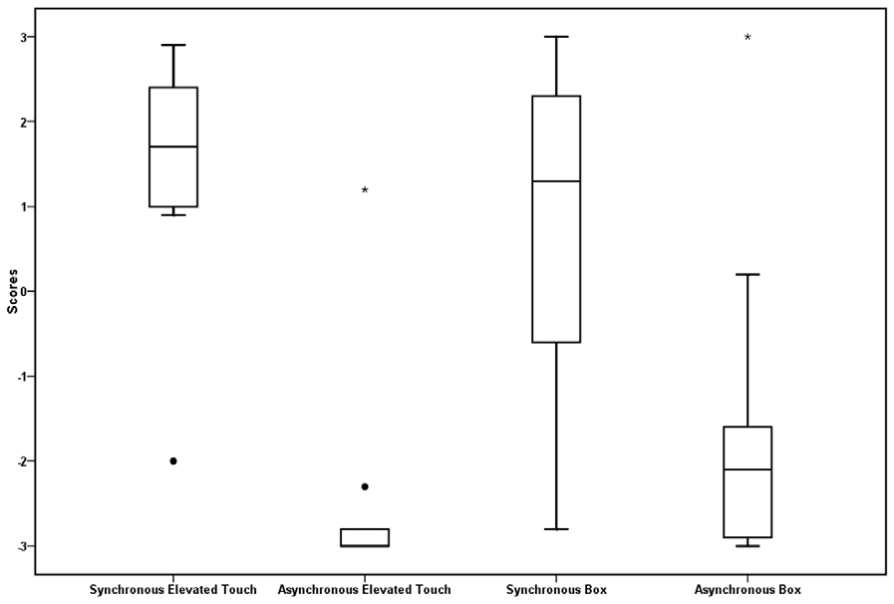
Box plots for experiment 2. Box plots of scores for experiment 2, for the elevated touch and touch on a box for synchronous and asynchronous tapping.

Two illusions were tested in separate groups of participants. (i) An illusion of supernatural touch caused at a distance (i.e., touch sensation caused by a finger elevated off a rubber hand; see Figure 3c). (ii) An illusion of touch felt on a non-hand object, in this case a white cardboard box (see Figure 3d). Touch was conducted in silence and scores to answers were obtained in writing after each condition. A 2×2 mixed model ANOVA revealed a significant main effect of touch type, *F* (1, 19) = 51.71, *p*<.01, with participants reporting higher affirmative scores in the synchronous condition (*M* = 1.03, *SD* = 1.74) than in the asynchronous condition (*M* = −2.06, *SD* = 1.58) (Figure 7). (See Table 1 for the question used in this analysis). There was no main effect of condition or an interaction (*p's*>.05) indicating no difference in reported scores between the conditions. These results show that participants scored the presence of these unusual experiences higher during synchrony than during asynchrony. In the case of the experience of touch caused by an elevated finger, participants offered highly vivid answers to open-ended questions about their experiences, often formulated in supernatural terms (see Table 2).

**Table 2:** Answers to open-ended questions of experiences of strangeness in experiment 2: supernaturally caused (elevated) touch.

Examples of answers to open-ended questions
“There are opposed magnets on the finger and the skin”
“It's a magnetic field impacting on my arm”
“It is witchy”
“It is black magic”
“There is an invisible extension on the finger”
“It's ESP”
“It's telekinetic”
“A magician makes my muscles contract”
“A force field is pressed onto my arm”
“There is invisible gel between the finger and my arm”

### Experiment 3: Touch Felt on Box with No Prior Onset of the Basic Illusion

In Experiment 2 we found, contrary to recent findings, an illusion of touch felt on a non-hand object like a box. We reasoned that this may have been due to the onset period for the basic illusion and therefore we next tested the hypothesis that the illusion would not arise without such prior onset. A paired samples t-test revealed no significant differences between participant scores in synchronous touch versus asynchronous touch (*p*>.05) (see Table 1 for the question used in this analysis). This suggests that without prior onset of the basic illusion, it is more difficult to experience the illusion of touch on a non-hand object like a cardboard box.

In experiments 2 and 3 there were no significant differences in participant reports, (*p's*>.05) in the control questions between the two touch conditions so the data were excluded from further analysis.

### Temperature Data

In experiments 2 and 3, temperature measurements were averaged to obtain a single temperature measurement for each participant for each tapping condition. The data for each experiment were analysed using paired samples t-tests, comparing synchronous tapping data to asynchronous tapping data. There was no significant difference in temperature in any of these experiments, all *p*s>.05. This suggests that temperature changes, while useful for distinguishing the basic RHI [10] and the version of the RHI used here, is not useful for distinguishing these more unusual experiences.

## Discussion

There was support for the primary hypothesis that a rubber hand illusion occurs during synchronous touch but not asynchronous touch in the basic set up. Further, that there will be odd and unusual experiences of touch felt on a non-hand box and supernaturally caused (elevated) touch during synchronous touch after illusion onset, as opposed to asynchronous touch. There was support for the secondary hypothesis that the odd experience of feeling a touch as if on a cardboard box would not arise without prior onset of the basic illusion.

The principal finding from experiment 1 is that this paradigm is useful for creating an illusion of touch on a foreign limb that appears visually aligned with one's own limb in personal space, and as produced by the finger touching the foreign limb. This illusion arises during synchronous rather than asynchronous touch and is robustly sustained during non-touch periods up to one minute. From experiment 2, the principal findings are that after onset of such an illusion, further sensory input tends to be incorporated into the illusion even if this requires that prior knowledge concerning bodily self-representation and the nature of causal relations be overridden. Specifically, the subsequent illusory experiences tend to override the prior knowledge that touch cannot be felt on a cardboard box and that touch is not delivered via supernatural causation involving invisible extensions of fingers, force-fields or telekinesis. The principal finding from experiment 3 is that without prior onset there is no significant difference between synchrony and asynchrony for touch felt on a cardboard box.

In experiment 2, new and unusual visuotactile input is presented to participants after illusion onset. Intuitively, this evidence should extinguish the illusion: how, after all, can the experience that a foreign rubber arm is the one that is being touched be correct if the arm is not in fact seen to be physically touched? How can a touch even begin to be felt on a box rather than something which at least looks like an arm? However, rather than extinguishing the illusion, participants incorporate the new evidence they have been presented with into the RHI.

The phenomena reported in experiment 2 also serve as indirect tests of the basic illusion created in this particular paradigm because the occurrence of these unusual experiential phenomena makes best sense on the assumption that the initial illusion is genuine and robust. If the rubber arm was not genuinely felt as the locus of touch and the experimenter's visible finger as the cause of the touch, then participants would not, for example, elaborate lack of physical contact in supernatural terms (See also [14], [15]).

### Temperature Changes

The temperature change of the participant's real arm, found in condition 2 of experiment 1 (Figure 4), replicates the intriguing finding by Moseley [10], obtained with the standard RHI. It adds to this finding by showing that the temperature effect does not arise exclusively because the foreign arm is known to be in a different location in personal space from the participant's own arm, rather it may arise because the foreign arm is somehow processed as non-self perhaps due to some complicated function of the touch sequences themselves. However, there is as yet no well-established explanation of this phenomenon (for discussion, see [16]). We use it here to demonstrate an objective difference between the two touch conditions, which is specifically suggestive of a basic type of RHI. We did not find any significant temperature changes in experiments 2 and 3. There may therefore be a difference in how skin temperature is modulated in the basic rubber hand illusion and in these more unusual variants. Further research is needed to determine the role and mechanism of these temperature changes in the standard RHI, our version of the RHI and unusual experiences within the RHI.

### Reconciling Conflicting Findings

Armel and Ramachandran [1] reported two startling somatosensory distortions in the RHI: touch was felt to a table top and touch was felt to a rubber hand outside of normal peripersonal space. Tsakiris and Haggard [6], [12] failed to replicate similar distortions when they tested whether the illusion would work for a non-hand rubber object. This has led to debate about the involvement of “bottom-up” and “top-down” processes in the RHI (even though the meanings of these terms are as yet somewhat unclear). On the basis of their findings, Armel and Ramachandran suggest it is mainly a bottom-up process, driven by Bayesian perceptual learning. On the basis of their findings, Tsakiris and Haggard suggest that there is top-down modulation of the illusion from prior body image knowledge.

The fact that Tsakiris and Haggard did not replicate an experience of touch on a non-hand rubber object could be due to not introducing this subsequent to onset of the basic RHI. Vice versa, the success of Armel and Ramachandran in creating these sensory phenomena could be due to introducing the table-touch and touch on a rubber hand in extrapersonal space after exposing participants to the RHI via (counterbalanced) synchronous and asynchronous touch, thereby somewhat raising the probability that touch is felt in a location away from the participant's real hand. Our experiment 3 support this analysis.

The ease with which odd perceptual phenomena can be induced, at least subsequent to basic illusion onset, in the present study challenges the presumption that a *robust* bodily self-representation or body-image plays a significant role for multisensory processing. In probabilistic terms this means that the internal model that represents visual body image and proprioceptive body schema decreases its probability in the face of ongoing solutions to visuotactile conflict. Thus, increasingly odd somatic, causal and tactile experiences can occur as the prior body representation is “explained away”. This account of our normally so stable body image is consistent with an elegant recent computational account of out-of-body experiences affecting bodily self-location. According to this account “online processing of body-related multisensory information in the brain is more like ongoing puzzle solving of which the normally experienced embodied self-location is just a fragile and only temporarily stable solution, which is a setting that is naturally suited for the Bayesian approach to sensory information processing” [17]. This overall approach seems to be consistent with that taken by Metzinger [7], [18] on phenomenal self-models. Further studies are needed to investigate the time course of such a possible explaining away effect (see Ref. [19] which reported increased somatosensory ERPs to tactile stimuli following training with synchronous but not asynchronous touch).

In the case of the illusion of touch to a non-hand object, it is also possible that recalibration of position sense during the initial rubber hand illusion period could lead to a remapping of touch and proprioception to the location of the seen touches on the foreign hand. When the image of the rubber hand is replaced with a white box, participants might experience the hand to be inside the box (and perhaps detached and partly invisible) and the touches being sensed through the box onto their hand. This explanation would also involve a degree of supernatural experience as it requires positing an invisible own arm extending through the wall of the box. More generally, if this explanation applies to illusory touch felt near an arm that is experienced to be detached or invisible, then it seems consistent with the idea that the visual body image is explained away and plays a decreasing role in multisensory integration after illusion onset.

The fact that prior induction of the basic rubber hand illusion is necessary for the illusion for the non-hand object seems consistent with earlier claims that the causal mechanisms of the rubber hand illusion involves multisensory integration in near-personal space [20], [21], performed by neuronal populations in multisensory areas (premotor cortex and posterior parietal cortex; [20], [22], [23], [24] Thus for tactile signals to be remapped to the rubber hand in the standard RHI, and for a drift in proprioception to occur, the rubber hand has to be placed in an anatomically congruent position [6], [22]). The fact that touch can be experienced on the white box only after the induction of the illusion with the rubber hand seems to be consistent with this view [21]. Thus bimodal visual-tactile cells in premotor and posterior parietal cortex [23], [24] could represent the multisensory stimuli in coordinates centered on the hand. Potentially this could also help explain why the illusion works in the elevated touch condition. The visual stimulus is close to the hand, that is within near-personal space [21] and it has been reported that many bi-modal visual-tactile cells in premotor cortex respond to objects presented within 30 cm from the hand [25]. A prediction of this model [21] would be that the illusion of elevated touch would not work if the finger was placed further than 30 cm from the hand. Note that, though this framework could yield a contributing or necessary factor for the illusion, it is not sufficient given that there is very little tendency for the illusion to arise when this kind of visual stimulus occurs in asynchrony with the felt touch. Further, if participants' answers to the open-ended questions (Table 2) are to be trusted, they perceive that something like an invisible extension of the finger is causing a touch on the rubber hand in the elevated touch condition, suggesting that this illusion has a very strong element of causal inference rather than merely touch mapped to near-personal space. Mapping in near-personal space thus seems tightly connected to causal inference.

### Wider Significance of Supernatural Experiences

Participants experience these distortions as very strange and weird, and the results show that supposedly normal and healthy volunteers still wonder if there is in fact a touch felt on a box, or being caused by an invisible extension of a moving finger. However, they do not truly believe that their arm has radically changed in appearance, or that a magician is really making their muscles contract. We speculate that the experience is not elevated to full-blown belief because participants very well know that they could perform a disconfirming reality test on their experience, if only the experimenter would allow them to take off the head-mounted display or move their arm around freely. It is noteworthy however, that, in response to such unusual visuotactile ambiguity, healthy participants volunteer supernatural explanations that they normally would never entertain, not even as a remote possibility. This may be relevant for theories of delusion formation because it shows that even psychiatrically healthy individuals may readily resort to supernatural explanations of unusual low-level sensory experiences [26], [27]. This gives some support for one-deficit theories of delusions according to which delusions arise as normal responses to unusual experiences [28]. In particular, it seems that a second deficit of rationality [29] may not be needed for these rather bizarre experiences to arise. We speculate that the belief state would evolve to a full-blown delusion if the sensory mis-integration was more persistent and if reality-testing for the ensuing unusual experience was more chronically unavailable. The transition from unusual experience to delusional belief would be facilitated if, as our results suggest, body image can be explained away such that it is merely a fragile and only temporarily stable solution, which is hostage to the probabilistic workings of sensory integration rather than the other way around.
